# East/Central/South African Genotype Chikungunya Virus, Brazil, 2014

**DOI:** 10.3201/eid2105.141727

**Published:** 2015-05

**Authors:** Maria G. Teixeira, Alcina M.S. Andrade, Maria da Conceição N. Costa, Jesuína S.M. Castro, Francisca L.S. Oliveira, Cristina S.B. Goes, Maricelia Maia, Eloisa B. Santana, Bruno T.D. Nunes, Pedro F.C. Vasconcelos

**Affiliations:** Instituto de Saúde Coletiva da Universidade Federal da Bahia, Salvador, Brazil (M.G. Teixeira, M.C.N. Costa);; Secretaria da Saúde do Estado da Bahia, Salvador (A.M.S. Andrade, J.S.M. Castro);; Secretaria da Saúde de Feira de Santana, Feira de Santana, Brazil (F.L.S. Oliveira, C.S.B. Goes, M. Maia, E.B. Santana);; Instituto Evandro Chagas, Ananindeua, Brazil (B.T.D. Nunes, P.F.C. Vasconcelos);; Universidade do Estado do Pará, Belém, Brazil (P.F.C. Vasconcelos)

**Keywords:** Chikungunya virus, African genotype, Brazil, viruses, zoonoses, arboviruses, vector-borne infections, alphaviruses, *Suggested citation for this article*: Teixeira MG, Andrade AMS, Costa MCN, Castro JSM, Oliveira FLS, Goes CSB, et al. East/Central/South African genotype chikungunya virus, Brazil, 2014 [letter]. Emerg Infect Dis [Internet]. 2015 May [*date cited*]. http://dx.doi.org/10.3201/eid2105.141727

**To the Editor:** Chikungunya virus (CHIKV) is an arthropod-borne alphavirus (family *Togaviridae*) comprising 3 genotypes: West African, East/Central/South African, and Asian (*1*). This zoonotic pathogen originated in Africa and since 2004 has caused outbreaks in several countries on different continents (*2*). In 2013, CHIKV reached the Americas and caused an explosive epidemic that has already caused 1,231,077 cases in 43 countries (*3*).

In Brazil, autochthonous cases of chikungunya were confirmed in September 2014 in Feira de Santana (FSA), a city of 612,000 residents (*4*) near the eastern edge of Bahia State in east-central Brazil. Surprisingly, the CHIKV genotype was determined to be East/Central/South African and not the Asian genotype that is circulating in the Americas; this finding was based on sequence data obtained from a cell culture viral isolate using an Ion Torrent platform (*3*,*5*).

Dengue is endemic/epidemic to FSA, and the first cases of chikungunya were mistakenly reported as dengue. Beginning in July 2014, when dengue virus transmission is low, an increased number of suspected cases of dengue from a FSA neighborhood caught the attention of local surveillance officials. CHIKV infection was suspected because results of laboratory tests for dengue (nonstructural 1 and IgM ELISA) were negative, and the patients complained mainly of high fever and intense bilateral joint pain accompanied by swelling (*4*). IgM ELISA and quantitative reverse transcription PCR conducted at the Instituto Evandro Chagas (Ananindeua, Brazil) confirmed the cause of illness as CHIKV. The sequences obtained in this study were deposited in the GenBank under accession nos. KP164567–KP164572.

Data from epidemiologic investigations suggested that the index case-patient could have been a Brazilian citizen living in Luanda, Angola, who visited his family in FSA. He went to an emergency health unit in FSA on May 28, reporting intense joint pain and high fever. His laboratory tests (nonstructural 1 and IgM) for dengue were negative. On June 4 (epidemiologic week [EW] 23), another person sought care for similar symptoms, and new cases emerged, all in residents in that same neighborhood (*4*).The epidemic peaked in EW 39, when 200 cases were reported. Cases then decreased, and in EW 48 only 10 cases were reported (Figure).

**Figure:**
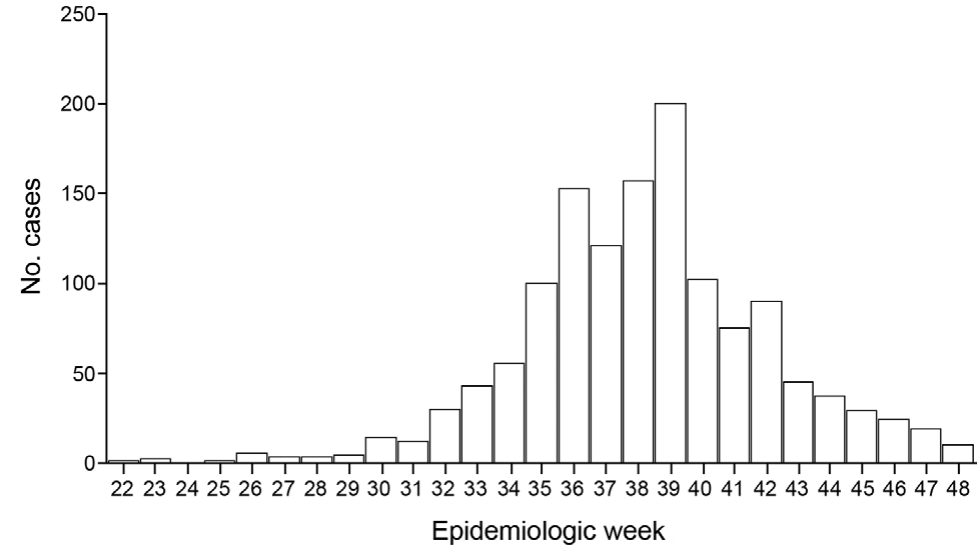
Reported cases of chikungunya fever, by epidemiologic week. Feira de Santana, Bahia State, Brazil, 2014.

In FSA, of the 1,346 chikungunya cases (219.9/100,000 residents) reported through EW 48, a total of 52.4% (1,498.1/100,000) patients lived in the same neighborhood as the index patient. However, the other 77 neighborhoods in FSA also recorded cases. Twice as many cases occurred among female patients (67.1% of cases) as among male patients. All age groups were affected; incidence was highest in persons 20–49 years of age (56.2%; 267.1 cases/100,000 inhabitants). The main clinical manifestations were high fever, arthralgia and arthritis with edema, headache, myalgia, rash, and itching. As of EW 48, no deaths were recorded (*4*).

CHIKV is transmitted by *Aedes aegypti* and *Ae. albopictus* mosquitoes, but FSA has *Ae. aegypti* only, and the Premise Index was 1.1% on January 2014 (*6*). Thus, during EW 36, the surveillance service of FSA began intense actions to combat that vector (*5*) by using integrated environmental management (*7*): elimination of breeding sites, applications of larvicide in water bodies, spraying insecticide (ultra-low volume), mobilization, and community education. However, cases continued to be diagnosed in neighborhoods in FSA, and transmission was detected in another municipality 77 km from FSA (391 cases through EW 48). Isolated cases imported from FSA were detected in other municipalities of Bahia State (*8*).

This epidemic had some unusual aspects. First, it was not caused by the Asian genotype circulating in affected countries of the Americas, which maintain intense tourism and trade with Brazil. Second, it occurred during the dry season, when little dengue transmission was occurring. The introduction of a person from a country reporting CHIKV activity (*9*) into an area infested by *Ae. aegypti* mosquitoes and having a population immunologically naive to CHIKV created favorable conditions to establish a local transmission cycle with quick production of many cases.

Concurrently with the outbreak in FSA, chikungunya cases were detected in Oiapoque municipality (*10*), Amapá State (northern Brazil bordering French Guiana); these cases were caused by the Asian genotype (genotype determined by nearly complete genome sequencing using an Ion Torrent sequencer). The picture so far suggests that expansion of the epidemic to other places in Brazil can be caused both by internal movement of persons and by new cases imported from other countries.

Chikungunya fever is a health problem that threatens Brazilian society and poses a challenge for health authorities. CHIKV produces epidemics of great magnitude, is highly debilitating, and does not have any specific treatment or vaccine. This situation is creating serious social and economic consequences for low- and middle-income countries because of the excessive demand on health services and the social security programs used by much of the population. Therefore, the global spread of chikungunya fever highlights the need to mobilize national and international efforts to focus scientific research on developing tools to prevent this disease.
